# Exploring the relationship between population density and maternal health coverage

**DOI:** 10.1186/1472-6963-12-416

**Published:** 2012-11-21

**Authors:** Michael Hanlon, Roy Burstein, Samuel H Masters, Raymond Zhang

**Affiliations:** 1 Institute for Health Metrics and Evaluation, University of Washington, 2301 5th Avenue, #600, Seattle, WA, 98121, USA; 2 Feinberg School of Medicine, Northwestern University, Seattle, USA

**Keywords:** Population density, Obstetric health coverage, Maternal and child health

## Abstract

**Background:**

Delivering health services to dense populations is more practical than to dispersed populations, other factors constant. This engenders the hypothesis that population density positively affects coverage rates of health services. This hypothesis has been tested indirectly for some services at a local level, but not at a national level.

**Methods:**

We use cross-sectional data to conduct cross-country, OLS regressions at the national level to estimate the relationship between population density and maternal health coverage. We separately estimate the effect of two measures of density on three population-level coverage rates (6 tests in total). Our coverage indicators are the fraction of the maternal population completing four antenatal care visits and the utilization rates of both skilled birth attendants and in-facility delivery. The first density metric we use is the percentage of a population living in an urban area. The second metric, which we denote as a density score, is a relative ranking of countries by population density. The score’s calculation discounts a nation’s uninhabited territory under the assumption those areas are irrelevant to service delivery.

**Results:**

We find significantly positive relationships between our maternal health indicators and density measures. On average, a one-unit increase in our density score is equivalent to a 0.2% increase in coverage rates.

**Conclusions:**

Countries with dispersed populations face higher burdens to achieve multinational coverage targets such as the United Nations’ Millennial Development Goals.

## Background

It has been recognized that some social services are more easily delivered to concentrated populations [1]. This argument has been implicitly applied to health services by two groups of researchers. The first group has used population density as an independent variable in analyses of coverage and outcomes. Studies of aggregated populations often incorporate population density as a continuous variable [2-4], while patient-level studies typically account for density as a binary, patient-level characteristic [5,6]. Generally, this literature includes population density as an afterthought, rather than a determinant of interest. A second group of researchers has estimated the effect of distance or travel time on service utilization [7-11]. This literature consistently finds lower distances or travel times increase the utilization of some services. Yet travel times are a function of many factors, including how a population is distributed across space. Holding health system resources constant, a denser population is expected to face lower travel times than a dispersed population. So given the conclusions from the travel time literature, we hypothesize population density is an underlying determinant of some coverage rates.

An exhaustive test of this hypothesis would require data on coverage rates, population density, road networks and facility locations over time. To our knowledge, these data does not exist for any single country, let alone across countries. In this analysis, we conduct a cross-country, cross-sectional analysis of population density and coverage levels of three maternal health services. This is the first test of this relationship at a national level. We believe it is important because of the implications for achieving multinational coverage targets such as the United Nations’ Millennial Development Goals (MDGs) [12]. To the degree population density matters, countries with dispersed populations face higher burdens to achieve uniform targets like the MDGs.

## Methods

We execute a cross-sectional analysis of 178 country-level observations. This is represented by equation (1), in which the subscript *c* denotes a country-specific variable. To hold the health system’s resources constant, we include per capita health expenditure and the number of hospital beds per 1,000 people in the country. We further include the total fertility rate and the number of four-wheel vehicles per capita as determinants of demand. The hypothesis under consideration predicts *β*_1_ > 0.

(1) $$coverage_{c}=\beta_{0}+\beta_{1}\;density_{c}+\beta_{2}\;In(health\;expenditure)+\beta_{3}(hospital\;beds_{c})+\beta_{4}(total\;fertility\;rate_{c})+\beta_{5}(four-wheel\;vehicles_{c})+\varepsilon_{c}$$

We use three different measures from 2009 as coverage variables: (*i*) the percentage of pregnant women who complete four antenatal care visits prior to birth; (*ii*) the percentage use of a skilled birth attendant at delivery; and (*iii*) the percentage use of in-facility delivery services. These data series were produced by the Institute for Health Metrics and Evaluation (IHME) [13]. We chose IHME’s series because it is (to our knowledge) the most complete source of coverage estimates for these services at the national level. For health spending, we use the natural log of total per-capita health expenditure for 2009, as reported by the World Health Organization in real 2009 US dollars [14]. For the number of four-wheel vehicles, hospital beds and total fertility rate, we use data series from IHME [13]. These independent variables are contemporaneous with the dependent variable because we expect little-to-no time lag in their effect on coverage levels.

A challenge with using population density is in identifying an appropriate metric to represent the concept. Prevailing density metrics may be inappropriate covariates in a cross-country analysis for a variety of reasons. For example, an unadjusted ratio of population-per-area at the national level includes uninhabited territory within a country’s borders. While those “empty spaces” may be relevant to some analyses, they are mostly irrelevant to analyses of health service provision (service is not provided in areas without people). Therefore, the failure to discount uninhabited areas downwardly biases the population-per-area ratio in some countries. Metrics like the percentage of the population residing in an “urban” or “dense” area are more appropriate to an analysis of health service provision, but the definitions of “urban” and “dense” can differ across countries [15,16]. There are attempts to reconcile these differences, but many approaches excessively conflate the relationship between density with wealth [17]. So it is unclear if prevailing metrics are appropriate for a cross-country comparison, which may in part explain why so few analyses have considered population density’s effect on service provision [18].

To address this challenge, we separately use two measures of population density: (*i*) percentage of the population residing in an “urban” area; and (*ii*) a novel metric designed for this analysis, which we denote as a country’s “density score.” All these metrics are calculated using data from the Global Rural–urban Mapping Project (GRUMP) Alpha Version, from the year 2000 [19,20]. We choose to use GRUMP data for three reasons. First, we required a single dataset to provide population data at a global level. Second, in contrast to a dataset like LandScan, GRUMP is freely available to all users. Third, GRUMP provided extremely granular estimates in a consistently-measured scale. This is in contrast to the Gridded Population of the World (GPW) dataset, which reported data at both higher and idiosyncratic levels of aggregation. For example, the GPW reports population and area by administrative unit, but the size of these units vary drastically across countries. In some countries vast tracts of unpopulated area were indistinguishable from population centers (Saudi Arabia’s territory was segmented into a mere thirteen units, most of which included both populous cities and large tracts of desert).

 Only cross-sectional population data from the year 2000 is available at our desired granularity, so we calculate our density metrics for the year 2000 only. This is not contemporaneous with our dependent variable, and it could be a limitation of the analysis if population densities changed radically during the decade. However, our assumption is that our metric of population density did not significantly change over this period of time, especially since it is calculated on a global scale. Moreover, this lag ensures density is predetermined in the data-generating process, which justifies our use of ordinary-least squares estimation. The GRUMP data we employ has a resolution of thirty arc seconds. Thirty arc seconds is approximately one square kilometer at the equator, and the area decreases as a grid’s location approaches the poles. The rate of decrease is governed by the cosine function. So the rate of decrease is initially slow, but then speeds up as grids approach the poles [21]. We do not believe this change in area is relevant to our analysis, for two reasons. As previously noted, population metrics can be distorted when populations are assigned to uninhabited areas. This incorrect assignment is most likely to occur at the equator because that is where grids are the largest. Yet even at the equator, grids are small enough (one square kilometer) that the magnitude of this problem is inherently limited. Also, metrics could be distorted if the grids were trivially small [22]. This is not a concern with this analysis because few consequential settlements exist above or below 65° latitude, let alone at the poles. This analysis is inherently global in perspective, and even for countries near the poles, the bulk of their population is measured in reasonably-sized grids of close to one square kilometer.

We employ GRUMP’s “unadjusted” population estimates, which differ from published UN population estimates. An “adjusted” version of GRUMP exists in which population values are scaled so that country-level totals match published UN estimates. We use the unadjusted data, for two reasons. First, in percentage terms, the discrepancies were small. Second, the adjusted data was exposed to an additional transformation which was not relevant to this analysis. Moreover, it is unclear from the documentation precisely how that transformation was executed. The differences in estimates are not relevant to our findings, in that the “adjusted” metrics produce the same results and lead to the same conclusions. Given the ambiguity of the adjustment process and its irrelevance to this analysis, we prefer the “unadjusted” metrics.

GRUMP identifies “urban” grids in the dataset. We use the population values assigned to those grids to calculate our first density metric, which is the percentage of the country’s total population residing in an urban area. However, GRUMP uses satellite imagery of nightlights to identify urban areas, which is potentially problematic because nightlight usage has been shown to be strongly influenced by wealth [23,24]. This is an issue for this analysis because we attempt to identify density’s effect independently of wealth. So while this metric is generally accepted, it is unclear *a priori* how appropriate its usage is in this analysis. This concern leads us to generate country-level density scores.

To calculate a density score, we first use the GRUMP data to determine global density decile thresholds, such that each density decile contains 10% of the global population. Then for each country, we determine the percentage of the population residing in each global decile. In other words, we sum the population of each grid in a country assigned to each global decile, and then divide by the country’s total population. This is represented by equation (2), in which *d* and *pop*_*c*,*d*_ denote the decile and the corresponding percentage of the population from country *c* in that decile. For each country, we calculate a weighted average in which the weights increase along with the decile, per equation (3). This weighted average is a country’s density score.

(2) $$pop_{c,d}=\frac{\mathrm{population}\;\mathrm{residing}\;\mathrm{in}\;\mathrm{grids}\;\mathrm{with}\;\mathrm{density}\;\mathrm{belonging}\;\mathrm{to}\;\mathrm{decile}\;d}{\mathrm{total}\;\mathrm{population}\;\mathrm{of}\;\mathrm{country}\;c}$$

(3) $$score_{c}=\left(\sum_{d-1}^{10}\left(\frac{d-1}{9}*pop_{c,d}\right)\right)*100$$

Our method to calculate a density score is conceptually similar to the procedure outlined by Craig (1984), who examined population density data in Great Britain [25]. Given these scores are based on global density deciles, it is effectively a relative ranking of density across countries. If a country’s entire population resides in the lowest global decile, its score is zero. If the entire population resides in the highest decile, its score is one hundred. If a country’s density mirrors the global density, such that one-tenth of its population resides in each decile, its score is fifty. We could have adopted a different quantile to generate the scores, such as using vigintiles or percentiles rather than deciles. Also, we could have adopted a different weighting scale, rather than the uniform discounting in equation (3). An infinite number of options exist, many of which would have a marginal impact on the distribution (although not the order) of the scores. Given our regression model is most sensitive to the order, this choice has limited impact on this study. However, it could impact the magnitude of the effect, and therefore any policy implications (see the Discussion section).

## Results

**Figure 1:**
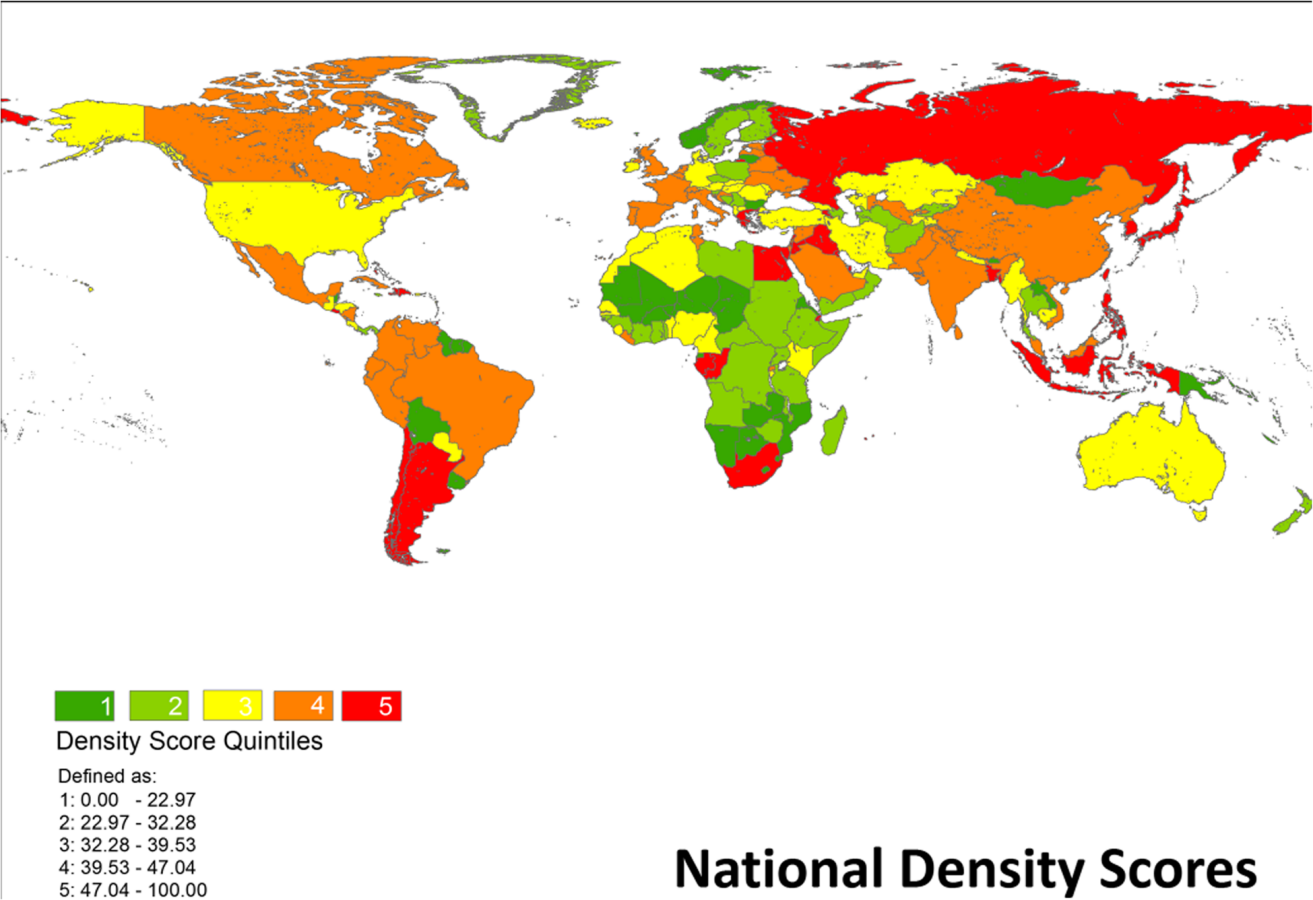
National Density Scores

Table 1 reports thresholds for global population density deciles for the year 2000, as calculated from GRUMP data. From this data, 50% of the global population resided in an area with a population density exceeding 564 persons per square kilometer. Figure 1 is national-level map of density scores by quintile. Several countries highlight how consequential our weighting process is to the results. Via an unadjusted calculation of population per area, Russia’s density is less than 10 persons per square kilometer and Egypt’s density is approximately 30 persons per square kilometer. By that metric, populations in both countries seem extremely dispersed. Yet once uninhabited areas are discounted, both countries emerge as being very dense because their populations are condensed in relatively small areas.

**Table 1:** Global population density deciles

***Decile***	***Population per km***^***2***^
1^st^	0 ≤ 49
2^nd^	49 ≤ 118
3^rd^	118 ≤ 220
4^th^	220 ≤ 368
5^th^	368 ≤ 564
6^th^	564 ≤ 849
7^th^	849 ≤ 1,321
8^th^	1,321 ≤ 2,433
9^th^	2,433 ≤ 5,201
10^th^	Over 5,201

Table 2 reports descriptive statistics of the variables used in the regression analysis (the webappendix reports all the data used in this analysis at the country level). Regression results with robust standard errors are published in Table 3. Across the six regressions, a total of 10 observations were clearly outliers but the results are robust to their exclusion. When comparing our two density metrics, we find the “percentage urban” variable consistently has a larger (and thus more significant) effect on coverage rates than the density score. However, when using the “percentage urban” as a predictor, the effect of health spending decreases. This may be due to the conflation of population density and wealth discussed in the Methods section. This concern is supported by the correlations in the density variables with health expenditure: it equals 0.73 for “percent urban” but decreases to 0.30 for the density score. This leads us to prefer the density score as the appropriate metric to represent density in this analysis.

**Table 2:** Descriptive statistics

**Variable**	**Mean**	**Standard deviation**	**Median**	**Skewness**
Antenatal visits (rate completing four)	0.693	0.218	0.787	−0.93
Skilled birth attendant (rate)	0.819	0.232	0.958	−1.28
In-facility delivery (rate)	0.793	0.249	0.941	−1.13
Percent population "urban"	52.8	24.6	55.2	−0.08
Density score	35.9	13.9	34.8	0.52
Log per capita health expenditure	6.0	1.4	6.1	0.00
Hospital beds, per 1000 population	3.7	3.0	2.7	0.97
Total fertility rate	2.9	1.5	2.4	0.90
Number 4-wheel vehicles, per capita	0.239	0.288	0.121	1.78

**Table 3:** Regression results

**Dependent variable**	**ANC4**		**SBA**		**IFD**	
**Density variable**	**% "urban"**	**score**	**% "urban"**	**score**	**% "urban"**	**score**
Density	0.003***	0.002**	0.004***	0.002**	0.004***	0.002*
	(0.001)	(0.001)	(0.001)	(0.001)	(0.001)	(0.001)
ln(health expenditure)	0.07***	0.101***	0.073***	0.115***	0.085***	0.129***
	(0.011)	(0.008)	(0.011)	(0.008)	(0.011)	(0.009)
Hospital beds, per capita	0.003	0.000	0.006	0.002	0.006	0.002
	(0.006)	(0.006)	(0.006)	(0.006)	(0.006)	(0.006)
Total fertility rate	−0.009	−0.009	0.000	0.002	0.002	0.003
	(0.011)	(0.011)	(0.010)	(0.010)	(0.010)	(0.011)
# 4-wheel vehicles, per capita	0.035	0.068	0.014	0.054	0.013	0.054
	(0.057)	(0.059)	(0.052)	(0.055)	(0.054)	(0.056)
Constant	0.130	0.039	0.154**	0.040	0.050	−0.058
	(0.066)	(0.070)	(0.062)	(0.068)	(0.064)	(0.070)

Point estimates with standard errors in parentheses below. The significance of the hypothesis test that the point estimate differs from zero is denoted by three stars at the 1% level; two stars at the 5% level and one star at the 10% level.

Across the three regressions which use the density score, the effects of per-capita expenditure and the density scores were significantly positive. The model has an untransformed dependent variable, but expenditure is included as a natural log. So the interpretation is that an increase of *e* (2.7182…) in expenditure would produce an increase of *β* in coverage. Point estimates ranged from 0.101 for antenatal visits to 0.129 to in-facility delivery. Therefore, on average, an increase in health expenditure per capita of 2.71 times is associated with a 12.9% increase in the use of in-facility delivery services. Regarding the density score, a one unit increase in a country’s score translates to a 0.2% increase in coverage for all three considered in this analysis. The number of hospital beds, the fertility rate and the number of vehicles failed to achieve statistical significance.

## Discussion

Increasing the log of health expenditure per capita by one unit had the effect of increasing coverage roughly 11% across the three interventions. This implies that increasing health expenditure per capita by 1% has the effect of increasing coverage by roughly 0.04%. In contrast, increasing the population density score by a single unit had the effect of increasing coverage by 0.2%. This comparison highlights that population density matters, but national policy makers’ ability to manage density is constrained. So as a practical matter, health expenditure is a more important determinant of interest. Yet these results suggest different countries face different challenges in realizing multinational targets like the MDGs. For example, Benin and Mali have similar per capita expenditure on health. However, Benin’s population is far denser than Mali’s, and Benin’s coverage rates are considerably higher. Population density may in part explain that difference.

Density scores could be valuable to some policy makers if they were calculated at a regional or national level, rather than a global level. For example, analysts focused on African societies could develop scores derived from African density deciles, or US states could be assigned density scores based on US population deciles. While policy makers do not control population density per se, they do control factors which are likely to affect it, such as road construction or building codes in developed countries. Ultimately, the appropriate choice is a function of the specific analysis under consideration. Yet we believe the approach described in this analysis is a methodological improvement over the use of prevailing measures of population density, and thus merits consideration from analysts interested in controlling for population density.

As noted in the Methods section, the magnitude (although not the direction) of the reported results could be contingent on the method used to calculate the scores. Consider a weighting scale which discounted differences between the lowest deciles and exaggerated them between the higher deciles. For clarity, *a priori* there is no sensible reason to adopt this strategy versus the one we employed. However, if an analyst’s objective was to exaggerate differences among the higher deciles, then an appropriate strategy might be to use the quadratic weight (^d-1^/_9_)^2^ rather than ^d-1^/_9_. This would skew the distribution of scores leftward (similarly, the square root of ^d-1^/_9_ would exaggerate the difference between the lowest deciles and skew the distribution rightward). Depending on the intervention under consideration, this skewness could impact (any likely impair) any statistical inference. It is important to note that skewness could lead to heteroskedasticity, and thus inefficient estimation [26]. In some cases, this could prevent an analyst from identifying an effect which exists in reality. Yet this example is not intended to suggest score distributions must necessarily be symmetric, or that exaggerating the difference between the lowest or highest deciles is universally inappropriate. Rather, the key point is that the metric’s construction might be relevant to some analyses, and analysts should consider those complexities when replicating our strategy to measure population density.

## Conclusion

This analysis examines how population density influences maternal health coverage. This is the first attempt to identify this relationship at a national level. We find that population density positively influences coverage, and the implications of this conclusion are significant for demographers, public health researchers and policy makers. Countries with low population densities face higher burdens to achieve coverage of some health services. Therefore, we predict those countries require more resources per capita to achieve multinational coverage targets such as the MDGs.

## Competing interests

The authors have no competing interests to declare.

## Authors′ contributions

MH devised the analytical strategy, including the notion of density scores, and authored the draft. RB processed geospatial data from GRUMP. SHM and RZ conducted the empirical analysis. All authors read and approved the final manuscript.

## Pre-publication history

The pre-publication history for this paper can be accessed here:

http://www.biomedcentral.com/1472-6963/12/416/prepub
